# Influence of Oleacein, an Olive Oil and Olive Mill Wastewater Phenolic Compound, on *Caenorhabditis elegans* Longevity and Stress Resistance

**DOI:** 10.3390/foods13132146

**Published:** 2024-07-05

**Authors:** Morgane Carrara, Myriam Richaud, Pierre Cuq, Simon Galas, Delphine Margout-Jantac

**Affiliations:** 1 Qualisud, Université de Montpellier, Avignon Université, CIRAD, Institut Agro, IRD, Université de La Réunion, 34093 Montpellier, France; delphine.margout-jantac@umontpellier.fr; 2 Institut des Biomolécules Max Mousseron (IBMM), UMR 5247, CNRS, ENSCM, Université de Montpellier, 34090 Montpellier, France; pierre.cuq@umontpellier.fr (P.C.); simon.galas@umontpellier.fr (S.G.)

**Keywords:** oleacein, *C. elegans*, aging, olive oil, olive mill wastewater

## Abstract

Oleacein, a bioactive compound of olive oil and olive mill wastewater, has one of the strongest antioxidant activities among olive phenolics. However, few reports explore the in vivo antioxidant activity of oleacein, with no clear identification of the biological pathway involved. Earlier studies have demonstrated a link between stress resistance, such as oxidative stress, and longevity. This study presents the effects of oleacein on *Caenorhabditis elegans* mean lifespan and stress resistance. A significant lifespan extension was observed with an increase of 20% mean lifespan at 5 µg/mL with a hormetic-like dose-dependent effect. DAF-16 and SIR-2.1 were involved in the effects of oleacein on the longevity of *C. elegans*, while the DAF-2 receptor was not involved. This study also shows the capacity of oleacein to significantly enhance *C. elegans* resistance to oxidative and thermal stress and allows a better understanding of the positive effects of olive phenolics on health.

## 1. Introduction

**Figure 1:**
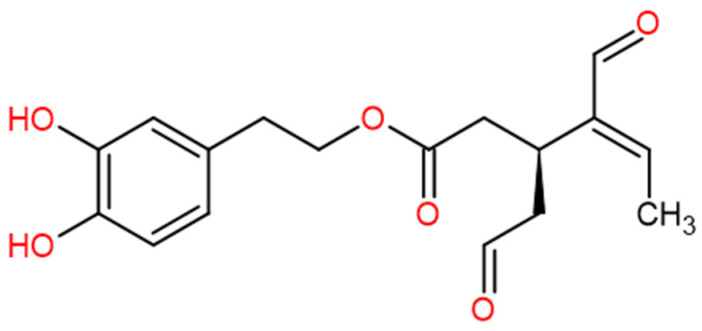
Oleacein structure.

Extra virgin olive oil (OO) comprises up to 36 phenolic compounds [1,2]; they have been associated with various health benefits [3,4], such as their antioxidant [5,6,7] and anti-inflammatory [8,9,10] activities, both in vitro and in vivo. Several studies also show that the consumption of OO phenolics may help to prevent cardiovascular, metabolic, or age-related diseases [11,12,13,14], and promote healthy aging [15,16]. In consequence, the consumption of OO is believed to be of great importance for the benefits of the Mediterranean diet [17]. Olive mill wastewater (OMW), an aqueous polluting waste generated during OO production, contains 98% of available olive phenolics [18], making it an interesting source of these compounds. Hydroxytyrosol is the most studied phenolic compound in OO and OMW for its antioxidant properties [19,20,21]. Oleacein (the dialdehydic form of elenolic acid conjugated with 3, 4-(dihydroxyphenyl)ethanol (3, 4-DHPEA-EDA)), another phenolic compound found in OO and OMW (Figure 1), has been the subject of fewer studies but has been shown to have one of the strongest in vitro antioxidant activities among olive phenolics [22,23,24,25]. Only a few studies on the in vivo testing of oleacein antioxidant activity are reported, with no clear identification of the biological pathways involved [26,27], although Nikou et al. suspect a contribution of the Ins/IGF-dependent pathway.

Lifespan is regulated by signaling pathways that are conserved from yeast to humans [28,29]. Some of the most widely studied are target of rapamycin (mTOR) signaling [30], nicotinamide adenine dinucleotide (NAD^+^)-dependent sirtuins [31], and insulin/insulin-like growth factor 1 (IGF-1) regulatory pathways [28]. The roundworm *Caenorhabditis elegans* (*C. elegans*) possesses all of these regulatory pathways [28]. Because of its short lifespan, and readily available molecular tools and mutant strains [32], *C. elegans* is a suitable model for drug screening [33,34]. Previous reports highlighted the positive effects of phenolic compounds on the lifespan of *C. elegans* [35,36,37]. For example, Brunetti et al. previously demonstrated the ability of hydroxytyrosol to increase *C. elegans* mean lifespan by 14% at 250 µg/mL [38]. 

It has been reported that some phenolic compounds may induce a hormetic response [39,40,41]. Hormesis is characterized by a moderate and non-permanent stress that induces a beneficial effect at low doses and a deleterious effect at high doses and is considered an adaptive response [42,43]. More precisely, hormetic compounds are a source of stress and at low doses trigger innate overcompensation with an overall positive effect. At higher doses, this stress overwhelms defense mechanisms, explaining the observed negative effect [44]. Previous reports have shown that the hormesis function is absent in *C. elegans* mutations in the *Daf-16 C. elegans* gene [45,46]. The *Daf-16* gene encodes an ortholog of the human FOXO family of transcription factors, and its genetic targets include, among others, reactive oxygen species (ROS) scavengers such as *Sod-3* (superoxide dismutase) or gene-encoding chaperones such as *Hsp-16.2* (heat shock protein) [47,48]. Activation/inhibition processes of the DAF-16 transcription factor are regulated by molecular pathways such as the *Daf-2* (Ins/IGF)-dependent signaling pathway. Downregulation of the *Daf-2* (Ins/IGF)-dependent signaling pathway leads to activation of DAF-16, resulting in a twofold extension of *C. elegans* lifespan [49,50]. The *C. elegans Sir-2.1* gene is an ortholog of mammalian SIRT1 that belongs to the *Sir-2* family of NAD^+^-dependent protein deacetylase-encoding genes. The deacetylase encoded by the *Sir-2.1* gene can extend *C. elegans* lifespan via the DAF-16 transcription factor [51], notably when activated by oxidative stress [52,53].

In this manuscript, we show for the first time that oleacein can promote healthy aging in an in vivo model of *C. elegans* by promoting its stress resistance and extending its lifespan expectancy. We also identify *C. elegans* genes involved in its mechanism of action.

## 2. Materials and Methods

Reagents and Chemicals

HPLC-MS-grade methanol and water were purchased from Carlo-Erba reagents (Carlo-Erba reagents, Val-de-Reuil, France). DMSO was purchased from Sigma-Aldrich (Sigma-Aldrich/Merck, Darmstadt, Germany). Oleacein was purchased from Toronto Research Chemicals (Toronto Research Chemicals, Toronto, ON, Canada, ref. O524550) and was dissolved in DMSO at 15 g/L and stored at −20 °C until use. The compound 5-fluorodeoxyuridine (FUdR) was purchased from Sigma-Aldrich (Sigma-Aldrich/Merck, Darmstadt, Germany) and was dissolved in water at 0.4 M and stored at −20 °C until use.

Oleacein stability in experimental media

S-base medium [54] with oleacein at 5 or 20 µg/mL was stored in the dark at 20 °C and regularly analyzed for up to 70 days. Oleacein was quantified by reverse-phase liquid chromatography (Ultimate 3000, Thermo Scientific, Waltham, MA, USA) coupled to quadripole mass spectrometry (ISQ EC, Thermo Scientific) and ESI in negative ion mode. Mobile phases were MeOH—0.001% formic acid (A) and water—0.001% formic acid (B) delivered at 0.4 mL/min with linear gradient elution across a biphenyl column at 40 °C. The gradient program was 0–2 min, 75% B; 2–10 min, 75–45% B; 10–13 min, 45% B; 13–14 min 45–0% B; 14–15 min 0% B; 15–16 min 0–75% B; and 16–20 min 75% B. The injection volume was 5 µL, and analyses were carried out in triplicate.

Worm Strains

The N2 Bristol strain was used as a wild-type genetic background. The following mutations were used in this study: GR1307 *daf-16* (*mgDf50)*; TJ356 *daf-16*::GFP *(zls356 [daf-16p::daf-16a/b::GFP* + *rol-6(su1006)]);* CB1370 *daf-2 (e1370)*; and VC199 *sir-2.1 (ok434)*, GC363 Bacteria *E. coli* HT115(DE3). All strains were provided by the CGC (Caenorhabditis Genetic Center—University of Minnesota), which is funded by NIH Office of Research Infrastructure Programs (P40 OD010440). Wild-type and mutant strains of *C. elegans* strains were grown on NGM lite^®^ plates (6 mm width) with *Escherichia coli* bacteria as a food source at 20 °C [55]. 

Lifespan assays

All lifespan assays were carried out as follows: 

*C. elegans* synchronized L4 larvae were placed in an ELISA 96 plate previously filled with S-base medium, with FUdR at 400 µM to avoid offspring, and heat-killed *E. coli* (HT115) as a food source. Increasing concentrations of oleacein or DMSO-only (solvent control) were added to the wells. Medium not supplemented with either oleacein or DMSO was used as an S-base control. These controls were used to determine whether DMSO alone has a significant impact on the lifespan of *C. elegans*.

The number of live nematodes were counted daily until they no longer responded to light touching with a platinum wire. Those killed by accidental manipulation were counted as censored data. XLSTAT-life statistical software v. 2021.1.1 (Addinsoft, New York, NY, USA) was used to plot survival data by the Kaplan–Meier method and differences between survival curves were calculated using the Log Rank test at a 95% confidence interval. All experiments were carried out at least three times with 50 individuals per modality.

DAF-16::GFP cellular localization

Synchronized TJ356 L4 larvae expressing the DAF-16::GFP fusion protein were placed in 15 mL falcon™ tubes filled with S-base containing 400 µM FUdR to avoid offspring, and heat-killed *E. coli* HT115 as a food source. Media were supplemented with 5 µg/mL oleacein or DMSO as a solvent control. The tubes were incubated for 3 days at 20 °C before examination by fluorescence microscopy (LEICA M205 FCA). Subcellular DAF-16::GFP localization for each nematode was classified as “cytosolic”, “intermediate”, or “nuclear”. The experiment was carried out three times with at least 150 nematodes per modality.

Resistance to heat-shock stress

Synchronized N2 L4 larvae were prepared as per the previous section. Following three days’ incubation at 20 °C, they were placed in a water bath at 35 °C to subject them to heat stress. After five hours under these conditions, the nematodes were left in medium at 20 °C overnight and then transferred to an NGM plate. Nematodes that did not respond to light touching with a platinum wire were noted as dead. The experiment was carried out three times with at least 150 individuals per modality.

Resistance to oxidative stress

The nematodes were prepared as per the two previous sections and incubated for three days at 20 °C. To remove oleacein, the samples were rinsed three times with S-base, and then incubated with 0.6 M hydrogen peroxide for 30 min to subject them to oxidative stress. The hydrogen peroxide was removed by rinsing three times with S-base. The nematodes were left in medium at 20 °C overnight and then transferred to an NGM plate. Those that did not respond to light touching with a platinum wire were noted as dead. The experiment was carried out three times with at least 150 individuals per modality.

## 3. Results

### 3.1. Oleacein Stability during Lifespan Trials in C. elegans

**Figure 2:**
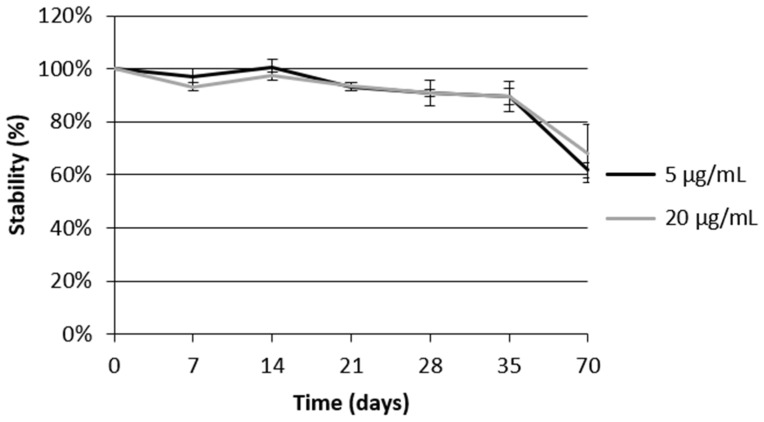
Oleacein stability in S-base at 20 °C.

Oleacein stability was monitored using UHPLC/MS analysis as it may undergo changes in concentration or chemical modification during survival assays. Figure 2 shows the evolution of oleacein concentration for up to 70 days in the S-base medium at a constant temperature of 20 °C. Stability was tested at 5 µg/mL and 20 µg/mL under the conditions as described above, and Figure 2 shows that oleacein was stable at both concentrations for 35 days, which is the maximal lifespan of most (N2) wild-type *C. elegans*.

### 3.2. Oleacein Effect on the Lifespan of (N2) Wild-Type C. elegans

**Figure 3:**
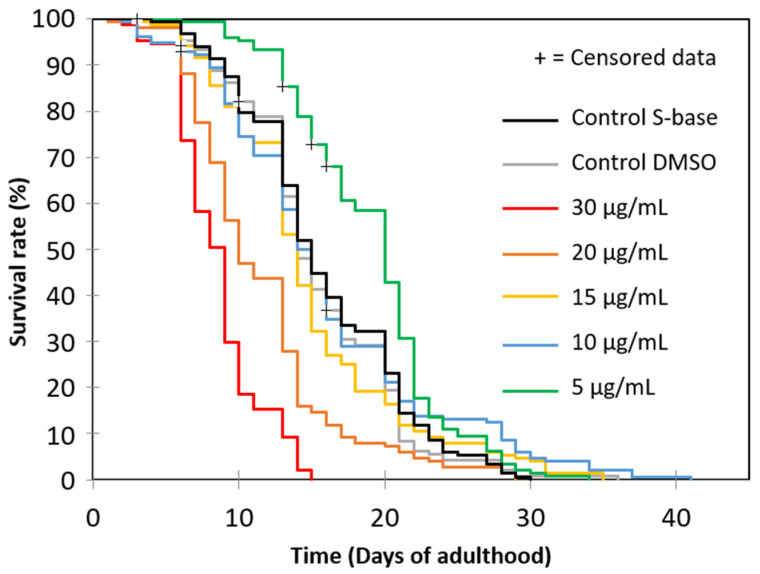
Oleacein impact on wild-type (N2) *C. elegans* lifespan.

To determine whether oleacein increases the longevity of *C. elegans*, an N2 wild-type strain of *C. elegans* adult nematodes was treated with increasing concentrations of oleacein (Figure 3, Table 1). Both S-base and DMSO controls correspond to, respectively, the medium conventionally used for maintaining nematode populations in liquid medium, and to the S-base medium supplemented with DMSO (cf. Section 2). As shown in Figure 3 and Table 1, mean lifespans were not significantly different between the two controls (LogRank *p* = 0.520), confirming that DMSO in the S-base does not have a significant impact on *C. elegans*. 

Treatment of the nematodes with the lowest oleacein concentration (5 µg/mL) increased longevity by up to 24%—mean life expectancy of 19.1 ± 0.4 days compared to S-base and DMSO control media with a mean life expectancy of 15.8 ± 0.4 and 15.4 ± 0.4 days, respectively; LogRank *p* < 0.0001 in both cases. Increasing oleacein concentrations to 10 or 15 µg/mL reduced the positive effect, and when exposed to even greater concentrations of oleacein, further significant reductions in longevity were observed compared to both S-base and DMSO control groups (Table 1). At 20 µg/mL, the mean lifespan was 11.5 ± 0.4 days and at 30 µg/mL it was 8.5 ± 0.2 days with LogRank *p* < 0.0001 in both cases. These findings indicate that oleacein has a hormetic-like dose-dependent effect on the longevity of *C. elegans*.

### 3.3. Oleacein Is More Effective Than Hydroxytyrosol in Extending the Longevity of (N2) Wild-Type C. elegans

**Figure 4:**
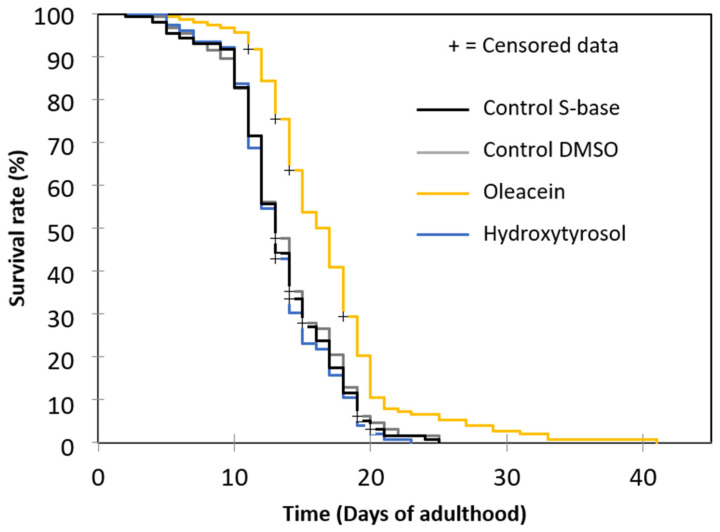
Comparative effects of oleacein and hydroxytyrosol treatment on wild-type *C. elegans* lifespan.

In a previous report, Brunetti et al. showed that hydroxytyrosol increases *C. elegans* longevity but at a considerably higher concentration (250 µg/mL) [38]. However, in this study, hydroxytyrosol at 5 µg/mL was compared to oleacein at the same concentration. As shown in Figure 4 and Table 2, as before, treatment with 5 µg/mL oleacein significantly increased the mean lifespan; however, the increase in lifespan with 5 µg/mL hydroxytyrosol was non-significant.

### 3.4. The Effect of Oleacein on the C. elegans Daf-16 Mutant

**Figure 5:**
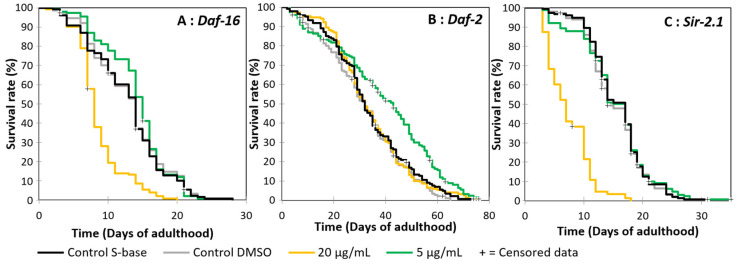
Effect of oleacein on *C. elegans* mutants’ lifespan.

As shown above (Figure 3), oleacein at higher concentrations has a converse effect by reducing worm lifespan expectancy in comparison to controls. Because of the involvement of the *C. elegans Daf-16* gene in hormesis and in longevity and stress resistance, particularly when boosted by phenolic compounds [56], the next step was to explore whether oleacein is involved in the *Daf-16*-dependent hormesis mechanism. To this end, the impact of oleacein on the lifespan of a *C. elegans* mutant for the *Daf-16* gene was investigated. As shown in Figure 5A and Table 3, treatment of nematodes with 5 µg/mL oleacein resulted in a lifespan comparable to both controls. Conversely, treatment with 20 µg/mL oleacein reduced the mean lifespan by 34% relative to control S-base and DMSO; this was an effect that was highly significant (LogRank *p* > 0.0001). These results suggest that the positive effect of oleacein on (N2) wild-type *C. elegans* is *Daf-16*-dependent.

### 3.5. Oleacein Effect on Nuclear Translocation of DAF-16 Transcription Factor

**Figure 6:**
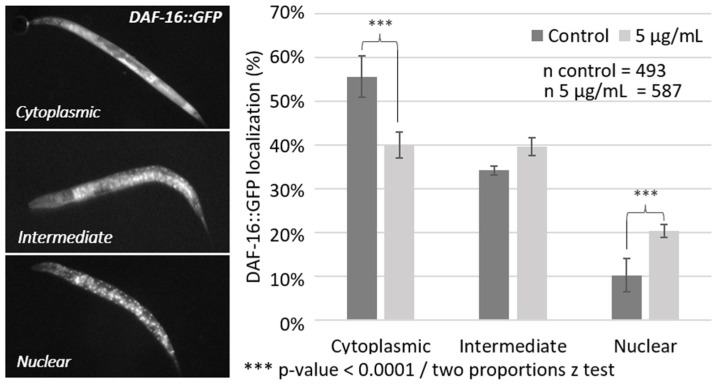
Effect of oleacein on DAF-16::GFP cellular localization.

In *C. elegans*, the transcription factor encoded by the hormetic gene *Daf-16* can be activated by various stress factors through its relocation to the nucleus from its position in the cytoplasm and activation of transcriptional targets. Therefore, the next step was to determine whether, in the absence of any stressor, oleacein could trigger translocation of the DAF-16 transcription factor from the cytoplasm to the nucleus. Such an effect may explain the positive effect of oleacein observed on *C. elegans* lifespan at low concentrations. Therefore, a *DAF-16::GFP* transgenic strain that allows direct observation by fluorescence was used (see Section 2). As shown in Figure 6, treatment with 5 µg/mL oleacein increased the nuclear translocation of DAF-16 by 10%, and reduced cytoplasmic localization by 15% compared to the DMSO control. 

### 3.6. Oleacein Effect on C. elegans Daf-2 Mutant

To further understand the ability of oleacein to activate DAF-16, the impact of oleacein on the lifespan of the *C. elegans Daf-2* mutant was studied using the protocols described previously. As shown in Figure 5B, and summarized in Table 3, exposing *Daf-2* mutant nematodes to 5 µg/mL oleacein significantly increased the mean lifespan by 21% compared to S-base and DMSO controls. Treatment with 20 µg/mL oleacein resulted in a lifespan comparable to controls. These results suggest that the positive effect of oleacein on wild-type (N2) *C. elegans* is independent of *Daf-2*, whereas the toxic effect could be countered by *Daf-2* downregulation. The DAF-16 transcription factor, which reduces oxidative stress, is constitutively active in *Daf-2* mutants. It may be supposed that oleacein toxicity at 20 µg/mL in the wild-type strain is related to an increase in oxidative stress. 

### 3.7. Oleacein Effect on C. elegans Sir-2.1 Mutant

Because the positive effect of oleacein on (N2) wild-type *C. elegans* is independent of *Daf-2*, and its negative effect may be related to increased oxidative stress, the next step was to study the possibility of oleacein DAF-16 activation being related to the deacetylase SIR-2.1 protein using a *Sir-2.1* mutant. As shown in Figure 5C and Table 3, 5 µg/mL oleacein resulted in a lifespan comparable to that of controls. This suggests that the positive effect of oleacein on (N2) wild-type *C. elegans* is SIR-2.1-dependent. Conversely, 20 µg/mL oleacein reduced the mean lifespan by almost 60% compared to controls; this was a difference that was highly significant—LogRank *p* > 0.0001. This negative effect seems to be greater on *Sir-2.1* mutants compared to the wild-type strain, and may be due to the poor resistance of *Sir-2.1* to oxidative stress, confirming that oleacein increases oxidative stress at higher doses. 

### 3.8. Oleacein Effect on C. elegans Resistance to Thermal and Oxidative Stress

**Figure 7:**
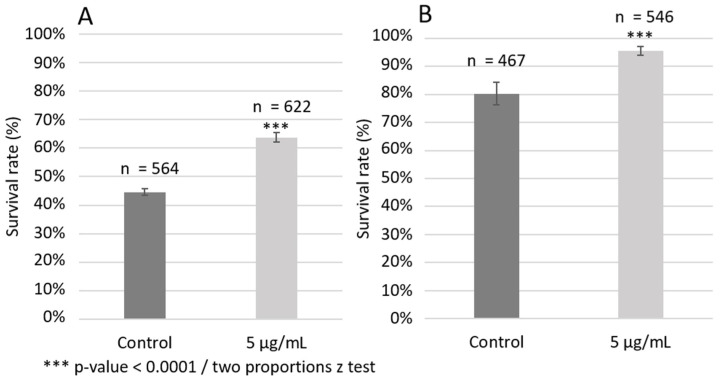
Effect of oleacein on wild-type *C. elegans* thermal (**A**) and oxidative stress (**B**) resistance.

Stress resistance is known to be strongly linked to *C. elegans* longevity [57]. To better understand this effect, the ability of oleacein to increase *C. elegans* resistance to thermal and oxidative stress was investigated. As shown in Figure 7A, 5 µg/mL oleacein increased the survival of wild-type nematodes by almost 20%, with a 63.7% survival rate after 5 h of exposure at 35 °C, compared to a 44.6% survival rate for the DMSO control. Moreover, as shown in Figure 7B, treatment with 5 µg/mL oleacein increased wild-type survival by 15% after 30 min of exposure to H_2_O_2_—95.6% survival as compared to 80.3% survival for the DMSO control. 

### 3.9. Biological Pathway Involved

**Figure 8:**
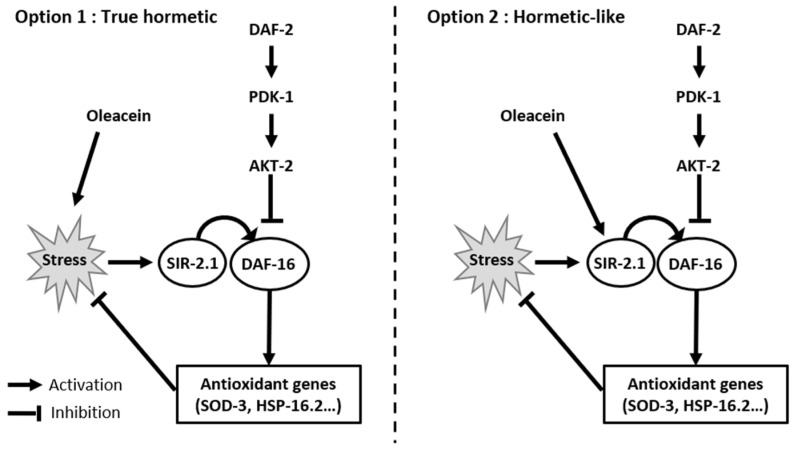
Possible mechanisms of action of oleacein.

In this study, oleacein had a hormetic-like dose-dependent effect on *C. elegans* longevity, with an increased lifespan at 5 µg/mL, no effect at 10 µg/mL and 15 µg/mL, and a reduced lifespan at 20 µg/mL and 30 µg/mL. Results showed that the low-dose positive effect of oleacein involved activation of the deacetylase SIR-2.1 protein, with subsequent activation of the transcription factor DAF-16. The negative effect of oleacein at 20 µg/mL or greater is probably related to increased oxidative stress. Based on these results, two mechanisms of action (Figure 8) may be proposed. 

One mechanism is that oleacein is a true hormetic compound and is a source of oxidative stress in vivo. As stated previously, hormesis is characterized by a treatment that induces a beneficial effect at low doses and a deleterious effect at high doses. At low doses of oleacein (5 µg/mL), oxidative stress triggers the activation of SIR-2.1 and DAF-16, which allows transcription of antioxidant proteins with an overall positive effect on longevity. At the medium doses of 10 and 15 µg/mL, oxidative stress induced by oleacein is compensated for by the nematodes’ defense mechanisms, resulting in an overall zero effect. The higher concentration of 20 µg/mL has a negative impact on longevity when the effect of SIR-2.1/DAF-16 is overcompensated by the oxidative stress induced by oleacein.

The second possibility is that oleacein is not a true hormetic compound and only has a hormetic-like dose-dependent effect on survival. In this scenario, oleacein is a direct activator of SIR-2.1/DAF-16, increasing survival rates of *C. elegans* at the lower concentration. The negative effect of oleacein at the higher concentration of 20 µg/mL acts in a parallel pathway of unknown origin. 

## 4. Discussion

The question of the hormetic nature of compounds has already been addressed for other phenolic compounds displaying the same dose–response effect, for example, quercetin or caffeic acid [58]. Many phenolic compounds were shown to increase *C. elegans* mean lifespan by 10 to 20% at concentrations of 18 to 250 µg/mL [58,59,60,61]. Other olive phenolics have shown similar activities, such as tyrosol which increased the mean lifespan by 21% at 35 µg/mL, or hydroxytyrosol which increased the mean lifespan by 14% at 250 µg/mL [38,62]. In a recent study, Cheng et al. showed that apigenin and chrysin could enhance *C. elegans* mean lifespan by, respectively, 23% at 10.8 µg/mL and 25% at 10.2 µg/mL [63]. It should be noted that in this study, oleacein at the lowest concentration of 5 µg/mL had a greater or similar impact on increasing lifespan than was previously reported for other phenolic compounds. Only Myricetin increased *C. elegans* lifespan to a significantly greater degree than oleacein (+33%), though at the considerably higher concentration of 160 µg/mL [64].

Together, these results—the first to identify the biological targets of oleacein—demonstrate the capacity of oleacein to increase lifespan and promote stress resistance in a *C. elegans* model, thereby contributing to the understanding of the beneficial effects of olive phenolics on health, in particular on healthy aging. Further experiments are necessary to explore the different mechanisms involved, especially regarding oxidative stress, though the findings of this study suggest oleacein is a true hormetic compound. Further studies on other in vivo models such as mice are needed to confirm this oleacein activity. Such explorations would contribute to a greater understanding of the action mechanisms involved in the activities of olive phenolic compounds and open avenues to their more effective and targeted exploitation in the future. 

## Figures and Tables

**Table 1:** Oleacein-dependent lifespan modulation of wild-type *C. elegans*.

Treatment	50th Percentile (Days)	Mean Lifespan (Days ± SD)	Max Lifespan (Days)	Code	Statistics (KM Analysis)	N (Censored)
					*p* = 0.520 (B)	
					*p* < 0.0001 (C)	
Control S-base	15	15.8 ± 0.4	30	A	*p* < 0.0001 (D) *p* = 0.401 (E) *p* = 0.375 (F) *p* < 0.0001 (G)	153 (1)
					*p* = 0.520 (A)	
					*p* < 0.0001 (C)	
Control DMSO	14	15.4 ± 0.4	36	B	*p* < 0.0001 (D) *p* = 0.530 (E) *p* = 0.256 (F) *p* < 0.0001 (G)	151 (3)
					*p* < 0.0001 (A)	
					*p* < 0.0001 (B)	
30 µg/mL	9	8.5 ± 0.2	15	C	*p* < 0.0001 (D) *p* < 0.0001 (E) *p* < 0.0001 (F) *p* < 0.0001 (G)	151 (0)
					*p* < 0.0001 (A)	
					*p* < 0.0001 (B)	
20 µg/mL	10	11.5 ± 0.4	29	D	*p* < 0.0001 (C) *p* < 0.0001 (E) *p* < 0.0001 (F) *p* < 0.0001 (G)	151 (0)
					*p* = 0.401 (A)	
					*p* = 0.530 (B)	
15 µg/mL	14	14.8 ± 0.5	35	E	*p* < 0.0001 (C) *p* < 0.0001 (D) *p* = 0.102 (F) *p* < 0.0001 (G)	154 (2)
					*p* = 0.375 (A)	
					*p* = 0.256 (B)	
10 µg/mL	15	15.9 ± 0.6	41	F	*p* < 0.0001 (C) *p* < 0.0001 (D) *p* = 0.102 (E) *p* = 0.007 (G)	153 (1)
					*p* < 0.0001 (A)	
					*p* < 0.0001 (B)	
5 µg/mL	20	19.1 ± 0.4	34	G	*p* < 0.0001 (C) *p* < 0.0001 (D) *p* < 0.0001 (E) *p* = 0.007 (F)	151 (3)

**Table 2:** Comparative effects of oleacein and hydroxytyrosol treatment on wild-type *C. elegans* lifespan.

Treatment	50th Percentile (Days)	Mean Lifespan (Days ± SD)	Max Lifespan (Days)	Code	Statistics (KM Analysis)	N (Censored)
			25		*p* = 0.577 (B)	
Control S-base	13	13.5 ± 0.3		A	*p* < 0.0001 (C)	158 (2)
					*p* = 0.504 (D)	
			25		*p* = 0.577 (A)	
Control DMSO	13	13.7 ± 0.3		B	*p* < 0.0001 (C)	155 (4)
					*p* = 0.238 (D)	
			41		*p* < 0.0001 (A)	
Oleacein 5 µg/mL	16	16.7 ± 0.4		C	*p* < 0.0001 (B)	160 (4)
					*p* < 0.0001 (D)	
			23		*p* = 0.504 (A)	
Hydroxytyrosol 5 µg/mL	13	13.3 ± 0.3		D	*p* = 0.238 (B)	154 (1)
					*p* < 0.0001 (C)	

**Table 3:** Effect of oleacein on *C. elegans* mutants’ lifespan.

Strain	Treatment	50th Percentile (Days)	Mean Lifespan (Days ± SD)	Max Lifespan (Days)	Code	Statistics (KM Analysis)	N (Censored)
				28		*p* = 0.711 (B)	
*Daf-16 (mgDf50)*	Control S-base	14	12.9 ± 0.4		A	*p* < 0.0001 (C)	153 (3)
						*p* = 0.128 (D)	
				28		*p* = 0.711 (A)	
*Daf-16 (mgDf50)*	Control DMSO	14	13.1 ± 0.4		B	*p* < 0.0001 (C)	151 (1)
						*p* = 0.291 (D)	
				20		*p* < 0.0001 (A)	
*Daf-16 (mgDf50)*	20 µg/mL	8	8.60 ± 0.28		C	*p* < 0.0001 (B)	152 (1)
						*p* < 0.0001 (D)	
				28		*p* = 0.128 (A)	
*Daf-16 (mgDf50)*	5 µg/mL	15	14.1 ± 0.4		D	*p* = 0.291 (B)	153 (1)
						*p* < 0.0001 (C)	
				74		*p* = 0.250 (B)	
*Daf-2 (e1370)*	Control S-base	32	33.8 ± 1.3		A	*p* = 0.916 (C)	148 (2)
						*p* = 0.0003 (D)	
				65		*p* = 0.250 (A)	
*Daf-2 (e1370)*	Control DMSO	32	31.8 ± 1.2		B	*p* = 0.382 (C)	152 (6)
						*p* < 0.0001 (D)	
						*p* = 0.916 (A)	
*Daf-2 (e1370)*	20 µg/mL	32	33.9 ± 1.2	76	C	*p* = 0.382 (B)	154 (2)
						*p* = 0.0003 (D)	
				76		*p* = 0.0003 (A)	
*Daf-2 (e1370)*	5 µg/mL	43	39.6 ± 1.6		D	*p* < 0.0001 (B)	155 (11)
						*p* = 0.0003 (C)	
				31		*p* = 0.544 (B)	
*Sir-2.1 (ok434)*	Control S-base	17	15.7 ± 0.4		A	*p* < 0.0001 (C)	153 (2)
						*p* = 0.811 (D)	
				31		*p* = 0.544 (A)	
*Sir-2.1 (ok434)*	Control DMSO	14	15.3 ± 0.4		B	*p* < 0.0001 (C)	149 (4)
						*p* = 0.454 (D)	
				18		*p* < 0.0001 (A)	
*Sir-2.1 (ok434)*	20 µg/mL	7	7.39 ± 0.30		C	*p* < 0.0001 (B)	151 (1)
						*p* < 0.0001 (D)	
				35		*p* = 0.811 (A)	
*Sir-2.1 (ok434)*	5 µg/mL	17	15.3 ± 0.5		D	*p* = 0.454 (B)	149 (5)
						*p* < 0.0001 (C)	

## Data Availability

The original contributions presented in the study are included in the article, further inquiries can be directed to the corresponding authors.

## References

[1] Parkinson L., Cicerale S. (2016). The Health Benefiting Mechanisms of Virgin Olive Oil Phenolic Compounds. Molecules.

[2] Celano R., Piccinelli A.L., Pugliese A., Carabetta S., di Sanzo R., Rastrelli L., Russo M. (2018). Insights into the Analysis of Phenolic Secoiridoids in Extra Virgin Olive Oil. J. Agric. Food Chem..

[3] Bendini A., Cerretani L., Carrasco-Pancorbo A., Gómez-Caravaca A.M., Segura-Carretero A., Fernández-Gutiérrez A., Lercker G. (2007). Phenolic Molecules in Virgin Olive Oils: A Survey of Their Sensory Properties, Health Effects, Antioxidant Activity and Analytical Methods. An Overview of the Last Decade. Molecules.

[4] Visioli F., Wolfram R., Richard D., Abdullah M.I.C.B., Crea R. (2009). Olive Phenolics Increase Glutathione Levels in Healthy Volunteers. J. Agric. Food Chem..

[5] Zhu L., Liu Z., Feng Z., Hao J., Shen W., Li X., Sun L., Sharman E., Wang Y., Wertz K. (2010). Hydroxytyrosol Protects against Oxidative Damage by Simultaneous Activation of Mitochondrial Biogenesis and Phase II Detoxifying Enzyme Systems in Retinal Pigment Epithelial Cells. J. Nutr. Biochem..

[6] Muriana F.J.G., la Paz S.M., Lucas R., Bermudez B., Jaramillo S., Morales J.C., Abia R., Lopez S. (2017). Tyrosol and Its Metabolites as Antioxidative and Anti-Inflammatory Molecules in Human Endothelial Cells. Food Funct..

[7] Wang W., Xia Y., Yang B., Su X., Chen J., Li W., Jiang T. (2017). Protective Effects of Tyrosol against LPS-Induced Acute Lung Injury via Inhibiting NF-κB and AP-1 Activation and Activating the HO-1/Nrf2 Pathways. Biol. Pharm. Bull..

[8] Aparicio-Soto M., Sánchez-Hidalgo M., Cárdeno A., González-Benjumea A., Fernández-Bolaños J.G., Alarcón-de-la-Lastra C. (2017). Dietary Hydroxytyrosol and Hydroxytyrosyl Acetate Supplementation Prevent Pristane-Induced Systemic Lupus Erythematous in Mice. J. Funct. Foods.

[9] Gutierrez M.B., Magiatis P., Nieto C.M.L. (2020). Use of Secoiridoids for the Treatment of Optic Neuritis.

[10] Vougogiannopoulou K., Lemus C., Halabalaki M., Pergola C., Werz O., Smith A.B., Michel S., Skaltsounis L., Deguin B. (2014). One-Step Semisynthesis of Oleacein and the Determination as a 5-Lipoxygenase Inhibitor. J. Nat. Prod..

[11] Xia M., Zhong Y., Peng Y., Qian C. (2022). Olive Oil Consumption and Risk of Cardiovascular Disease and All-Cause Mortality: A Meta-Analysis of Prospective Cohort Studies. Front. Nutr..

[12] Martínez-González M.A., Sayón-Orea C., Bullón-Vela V., Bes-Rastrollo M., Rodríguez-Artalejo F., Yusta-Boyo M.J., García-Solano M. (2022). Effect of Olive Oil Consumption on Cardiovascular Disease, Cancer, Type 2 Diabetes, and All-Cause Mortality: A Systematic Review and Meta-Analysis. Clin. Nutr..

[13] Boronat A., Serreli G., Rodríguez-Morató J., Deiana M., de la Torre R. (2023). Olive Oil Phenolic Compounds’ Activity against Age-Associated Cognitive Decline: Clinical and Experimental Evidence. Antioxidants.

[14] Jiménez-Sánchez A., Martínez-Ortega A.J., Remón-Ruiz P.J., Piñar-Gutiérrez A., Pereira-Cunill J.L., García-Luna P.P. (2022). Therapeutic Properties and Use of Extra Virgin Olive Oil in Clinical Nutrition: A Narrative Review and Literature Update. Nutrients.

[15] Foscolou A., Critselis E., Tyrovolas S., Chrysohoou C., Sidossis L.S., Naumovski N., Matalas A.-L., Rallidis L., Polychronopoulos E., Ayuso-Mateos J.L. (2019). The Effect of Exclusive Olive Oil Consumption on Successful Aging: A Combined Analysis of the ATTICA and MEDIS Epidemiological Studies. Foods.

[16] Fernández del Río L., Gutiérrez-Casado E., Varela-López A., Villalba J.M. (2016). Olive Oil and the Hallmarks of Aging. Molecules.

[17] Riolo R., De Rosa R., Simonetta I., Tuttolomondo A. (2022). Olive Oil in the Mediterranean Diet and Its Biochemical and Molecular Effects on Cardiovascular Health through an Analysis of Genetics and Epigenetics. Int. J. Mol. Sci..

[18] Roig A., Cayuela M.L., Sánchez-Monedero M.A. (2006). An Overview on Olive Mill Wastes and Their Valorisation Methods. Waste Manag..

[19] Kitsati N., Mantzaris M.D., Galaris D. (2016). Hydroxytyrosol Inhibits Hydrogen Peroxide-Induced Apoptotic Signaling via Labile Iron Chelation. Redox Biol..

[20] Mohan V., Das S., Rao S.B.S. (2016). Hydroxytyrosol, a Dietary Phenolic Compound Forestalls the Toxic Effects of Methylmercury-Induced Toxicity in IMR-32 Human Neuroblastoma Cells. Environ. Toxicol..

[21] Özbek N., Bali E., Karasu C. (2015). Quercetin and Hydroxytyrosol Attenuates Xanthine/Xanthine Oxidase-Induced Toxicity in H9c2 Cardiomyocytes by Regulation of Oxidative Stress and Stress-Sensitive Signaling Pathways. Gen. Physiol. Biophys..

[22] Nardi M., Bonacci S., De Luca G., Maiuolo J., Oliverio M., Sindona G., Procopio A. (2014). Biomimetic Synthesis and Antioxidant Evaluation of 3,4-DHPEA-EDA [2-(3,4-Hydroxyphenyl) Ethyl (3S,4E)-4-Formyl-3-(2-Oxoethyl)Hex-4-Enoate]. Food Chem..

[23] Paiva-Martins F., Fernandes J., Santos V., Silva L., Borges F., Rocha S., Belo L., Santos-Silva A. (2010). Powerful Protective Role of 3,4-Dihydroxyphenylethanol−elenolic Acid Dialdehyde against Erythrocyte Oxidative-Induced Hemolysis. J. Agric. Food Chem..

[24] Parzonko A., Czerwińska M.E., Kiss A.K., Naruszewicz M. (2013). Oleuropein and Oleacein May Restore Biological Functions of Endothelial Progenitor Cells Impaired by Angiotensin II via Activation of Nrf2/Heme Oxygenase-1 Pathway. Phytomedicine.

[25] Morelló J.-R., Vuorela S., Romero M.-P., Motilva M.-J., Heinonen M. (2005). Antioxidant Activity of Olive Pulp and Olive Oil Phenolic Compounds of the Arbequina Cultivar. J. Agric. Food Chem..

[26] Gutiérrez-Miranda B., Gallardo I., Melliou E., Cabero I., Álvarez Y., Magiatis P., Hernández M., Nieto M.L. (2020). Oleacein Attenuates the Pathogenesis of Experimental Autoimmune Encephalomyelitis through Both Antioxidant and Anti-Inflammatory Effects. Antioxidants.

[27] Nikou T., Liaki V., Stathopoulos P., Sklirou A.D., Tsakiri E.N., Jakschitz T., Bonn G., Trougakos I.P., Halabalaki M., Skaltsounis L.A. (2019). Comparison Survey of EVOO Polyphenols and Exploration of Healthy Aging-Promoting Properties of Oleocanthal and Oleacein. Food Chem. Toxicol..

[28] Kenyon C.J. (2010). The Genetics of Ageing. Nature.

[29] Château M.-T., Araiz C., Descamps S., Galas S. (2010). Klotho Interferes with a Novel FGF-Signalling Pathway and Insulin/Igf-like Signalling to Improve Longevity and Stress Resistance in *Caenorhabditis elegans*. Aging.

[30] Stanfel M.N., Shamieh L.S., Kaeberlein M., Kennedy B.K. (2009). The TOR Pathway Comes of Age. Biochim. Biophys. Acta.

[31] Imai S., Guarente L. (2014). NAD+ and Sirtuins in Aging and Disease. Trends Cell Biol..

[32] Johnson T.E. (2003). Advantages and Disadvantages of *Caenorhabditis elegans* for Aging Research. Exp. Gerontol..

[33] Artal-Sanz M., de Jong L., Tavernarakis N. (2006). *Caenorhabditis elegans*: A Versatile Platform for Drug Discovery. Biotechnol. J..

[34] Weinkove D., Zavagno G. (2021). Applying *C. elegans* to the Industrial Drug Discovery Process to Slow Aging. Front. Aging.

[35] Papaevgeniou N., Chondrogianni N. (2018). Anti-Aging and Anti-Aggregation Properties of Polyphenolic Compounds in *C. elegans*. Curr. Pharm. Des..

[36] Saul N., Pietsch K., Stürzenbaum S.R., Menzel R., Steinberg C.E.W. (2011). Diversity of Polyphenol Action in *Caenorhabditis elegans*: Between Toxicity and Longevity. J. Nat. Prod..

[37] Wilson M.A., Shukitt-Hale B., Kalt W., Ingram D.K., Joseph J.A., Wolkow C.A. (2006). Blueberry Polyphenols Increase Lifespan and Thermotolerance in *Caenorhabditis elegans*. Aging Cell.

[38] Brunetti G., Di Rosa G., Scuto M., Leri M., Stefani M., Schmitz-Linneweber C., Calabrese V., Saul N. (2020). Healthspan Maintenance and Prevention of Parkinson’s-like Phenotypes with Hydroxytyrosol and Oleuropein Aglycone in *C. elegans*. Int. J. Mol. Sci..

[39] Gems D., Partridge L. (2008). Stress-Response Hormesis and Aging: “That Which Does Not Kill Us Makes Us Stronger”. Cell Metab..

[40] Le Bourg E., Rattan S. (2008). Mild Stress and Healthy Aging: Applying Hormesis in Aging Research and Interventions.

[41] Rattan S.I.S. (2015). Hormetins as Novel Components of Cosmeceuticals and Aging Interventions. Cosmetics.

[42] Le Bourg E., Rattan S. (2014). Hormesis in Health and Disease.

[43] Mattson M.P. (2008). Hormesis Defined. Ageing Res. Rev..

[44] Calabrese E.J. (2008). Converging Concepts: Adaptive Response, Preconditioning, and the Yerkes–Dodson Law Are Manifestations of Hormesis. Ageing Res. Rev..

[45] Cypser J.R., Tedesco P., Johnson T.E. (2006). Hormesis and Aging in *Caenorhabditis elegans*. Exp. Gerontol..

[46] Cypser J.R., Johnson T.E. (2003). Hormesis in *Caenorhabditis elegans* Dauer-Defective Mutants. Biogerontology.

[47] Hartwig K., Heidler T., Moch J., Daniel H., Wenzel U. (2009). Feeding a ROS-Generator to *Caenorhabditis elegans* Leads to Increased Expression of Small Heat Shock Protein HSP-16.2 and Hormesis. Genes Nutr..

[48] Senchuk M.M., Dues D.J., Schaar C.E., Johnson B.K., Madaj Z.B., Bowman M.J., Winn M.E., Van Raamsdonk J.M. (2018). Activation of DAF-16/FOXO by Reactive Oxygen Species Contributes to Longevity in Long-Lived Mitochondrial Mutants in *Caenorhabditis elegans*. PLoS Genet..

[49] Kenyon C., Chang J., Gensch E., Rudner A., Tabtiang R. (1993). A *C. elegans* Mutant That Lives Twice as Long as Wild Type. Nature.

[50] Mukhopadhyay A., Oh S.W., Tissenbaum H.A. (2006). Worming Pathways to and from DAF-16/FOXO. Exp. Gerontol..

[51] Viswanathan M., Kim S.K., Berdichevsky A., Guarente L. (2005). A Role for SIR-2.1 Regulation of ER Stress Response Genes in Determining *C. elegans* Life Span. Dev. Cell.

[52] Berdichevsky A., Viswanathan M., Horvitz H.R., Guarente L. (2006). *C. elegans* SIR-2.1 Interacts with 14-3-3 Proteins to Activate DAF-16 and Extend Life Span. Cell.

[53] Wang Y., Tissenbaum H.A. (2006). Overlapping and Distinct Functions for a *Caenorhabditis elegans* SIR2 and DAF-16/FOXO. Mech. Ageing Dev..

[54] Stiernagle T., The C. elegans Research Community (2006). Maintenance of *C. elegans*. WormBook.

[55] Brenner S. (1974). The Genetics of *Caenorhabditis elegans*. Genetics.

[56] Liu L., Guo P., Wang P., Zheng S., Qu Z., Liu N. (2021). The Review of Anti-Aging Mechanism of Polyphenols on *Caenorhabditis elegans*. Front. Bioeng. Biotechnol..

[57] Zhou K.I., Pincus Z., Slack F.J. (2011). Longevity and Stress in *Caenorhabditis elegans*. Aging.

[58] Pietsch K., Saul N., Chakrabarti S., Stürzenbaum S.R., Menzel R., Steinberg C.E.W. (2011). Hormetins, Antioxidants and Prooxidants: Defining Quercetin, Caffeic Acid and Rosmarinic Acid-Mediated Life Extension in *C. elegans*. Biogerontology.

[59] Saul N., Pietsch K., Menzel R., Stürzenbaum S.R., Steinberg C.E.W. (2010). The Longevity Effect of Tannic Acid in *Caenorhabditis elegans*: Disposable Soma Meets Hormesis. J. Gerontol..

[60] Schlernitzauer A., Oiry C., Hamad R., Galas S., Cortade F., Chabi B., Casas F., Pessemesse L., Fouret G., Feillet-Coudray C. (2013). Chicoric Acid Is an Antioxidant Molecule That Stimulates AMP Kinase Pathway in L6 Myotubes and Extends Lifespan in *Caenorhabditis elegans*. PLoS ONE.

[61] Zheng S.-Q., Huang X.-B., Xing T.-K., Ding A.-J., Wu G.-S., Luo H.-R. (2017). Chlorogenic Acid Extends the Lifespan of *Caenorhabditis elegans* via Insulin/IGF-1 Signaling Pathway. J. Gerontol. A Biol. Sci. Med. Sci..

[62] Cañuelo A., Gilbert-López B., Pacheco-Liñán P., Martínez-Lara E., Siles E., Miranda-Vizuete A. (2012). Tyrosol, a Main Phenol Present in Extra Virgin Olive Oil, Increases Lifespan and Stress Resistance in *Caenorhabditis elegans*. Mech. Ageing Dev..

[63] Cheng Y., Hou B.-H., Xie G.-L., Shao Y.-T., Yang J., Xu C. (2023). Transient Inhibition of Mitochondrial Function by Chrysin and Apigenin Prolong Longevity via Mitohormesis in *C. elegans*. Free Radic. Biol. Med..

[64] Büchter C., Ackermann D., Havermann S., Honnen S., Chovolou Y., Fritz G., Kampkötter A., Wätjen W. (2013). Myricetin-Mediated Lifespan Extension in *Caenorhabditis elegans* Is Modulated by DAF-16. Int. J. Mol. Sci..

